# Brain physiome: A concept bridging *in vitro* 3D brain models and *in silico* models for predicting drug toxicity in the brain

**DOI:** 10.1016/j.bioactmat.2021.11.009

**Published:** 2021-11-12

**Authors:** Yoojin Seo, Seokyoung Bang, Jeongtae Son, Dongsup Kim, Yong Jeong, Pilnam Kim, Jihun Yang, Joon-Ho Eom, Nakwon Choi, Hong Nam Kim

**Affiliations:** a Brain Science Institute, Korea Institute of Science and Technology (KIST), Seoul, 02792, Republic of Korea; b Department of Bio and Brain Engineering, Korea Advanced Institute of Science and Technology (KAIST), Daejeon, 34141, Republic of Korea; c Next&Bio Inc., Seoul, 02841, Republic of Korea; d Medical Device Research Division, National Institute of Food and Drug Safety Evaluation, Cheongju, 28159, Republic of Korea; e Division of Bio-Medical Science & Technology, KIST School, Korea University of Science and Technology (UST), Seoul, 02792, Republic of Korea; f KU-KIST Graduate School of Converging Science and Technology, Korea University, Seoul, 02841, Republic of Korea; g School of Mechanical Engineering, Yonsei University, Seoul, 03722, Republic of Korea; h Yonsei-KIST Convergence Research Institute, Yonsei University, Seoul, 03722, Republic of Korea

**Keywords:** Brain physiome, *In vitro* 3D platform, Brain organoid, Brain-on-a-chip, *In silico* model

## Abstract

In the last few decades, adverse reactions to pharmaceuticals have been evaluated using 2D *in vitro* models and animal models. However, with increasing computational power, and as the key drivers of cellular behavior have been identified, *in silico* models have emerged. These models are time-efficient and cost-effective, but the prediction of adverse reactions to unknown drugs using these models requires relevant experimental input. Accordingly, the physiome concept has emerged to bridge experimental datasets with *in silico* models. The brain physiome describes the systemic interactions of its components, which are organized into a multilevel hierarchy. Because of the limitations in obtaining experimental data corresponding to each physiome component from 2D *in vitro* models and animal models, 3D *in vitro* brain models, including brain organoids and brain-on-a-chip, have been developed. In this review, we present the concept of the brain physiome and its hierarchical organization, including cell- and tissue-level organizations. We also summarize recently developed 3D *in vitro* brain models and link them with the elements of the brain physiome as a guideline for dataset collection. The connection between *in vitro* 3D brain models and *in silico* modeling will lead to the establishment of cost-effective and time-efficient *in silico* models for the prediction of the safety of unknown drugs.

## Highlights

• The brain physiome can be used to link 3D *in vitro* brain models and *in silico* models.
• *In vitro* 3D brain models recapitulate the brain physiome in an *in vitro* platform.
• *In vitro* 3D brain models can be used to predict adverse drug reactions in the brain.
• *In silico* models with *in vivo*-mimetic 3D brain models support the 3R principles.

## Graphical abstract

**Figure:**
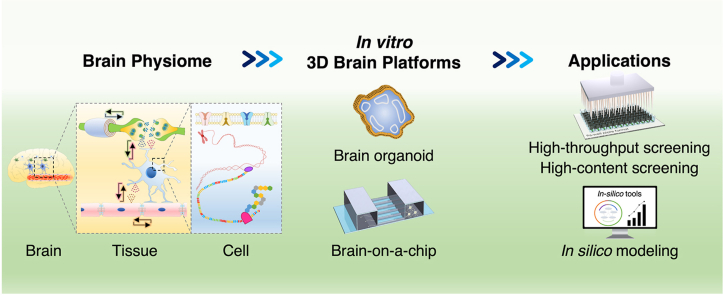

## Abbreviations

3Rs: Replacement, refinement, and reduction
Aβ: β-amyloid
AChE: Acetylcholinesterase
AD: Alzheimer's disease
ADME: Absorption, distribution, metabolism, and excretion
ASD: Autism spectrum disorders
Benz: Benzatropine
BBB: Blood–brain barrier
CCK: Cell counting kit
CiPA: Comprehensive *in vitro* proarrhythmia assay
CNS: Central nervous system
EC: Endothelial cell
ELISA: Enzyme-linked immunosorbent assay
EPSC: Excitatory postsynaptic current
GABA: γ-aminobutyric acid
HCS: High-content screening
HTS: High-throughput screening
iPSC: Induced pluripotent stem cell
IPSC: Inhibitory postsynaptic current
LPC: Lysophosphatidylcholine
MEA: Multi electrode array
MeCbl: Methylcobalamin
MG: Microglia
NMDA: *N*-methyl-d-aspartate
NMDAR: *N*-methyl-d-aspartate receptor
MTT: 3-(4,5-dimethylthiazol-2-yl)-2,5-diphenyl-2H-tetrazolium bromide
NO: Nitric oxide
OLG: Oligodendrocyte
OP: Organophosphate-based compound
PD: Parkinson's disease
PDMS: Polydimethyl siloxane
PNS: Peripheral nervous system
PS: Polystyrene
RT-PCR: Real-time polymerase chain reaction
SMEI: Severe myoclonic epilepsy in infancy
SMOTE: Synthetic minority oversampling technique
SVM: Support vector machine
ZO-1: Zonula occludens-1

## 1 Introduction

New biopharmaceuticals are constantly being developed for the treatment of severe disorders. However, the prediction of a drug's biological safety in humans is challenging because, for ethical and economic reasons, human-based trials cannot be used prior to the *bona fide* clinical trials [1,2]. Recently, *in silico* models have emerged as an approach to determine drug responses using computational tools. These models are powerful tools for the massive screening of drug candidates in a time-efficient and cost-effective manner. For the development of computational models, large amounts of experimental data, such as those concerning cellular and acellular responses to drug treatment, should be collected. However, because datasets obtained from individual studies pertain to various biological scales, it is difficult to use them directly for *in silico* modeling.

The physiome is a concept that can be used to organize data distributed on various biological scales. It is an integrated term that describes the functional behavior of a biological system. The elements of the physiome cover a broad range of physiological complexities, including the biochemical, biophysical, and anatomical environments of cells, tissues, and organs [3]. In this regard, the brain physiome is related to the physiological behavior of brain elements, in normal or abnormal states. Consideration of the brain physiome is crucial for determining drug-mediated responses in the brain tissue. Upon drug administration, drug metabolites transported to the brain can alter cellular behavior and acellular microenvironment therein, in some cases leading to mental or behavioral dysfunction if they are not sufficiently safe for use. Understanding the brain physiome can aid in the analysis of a large dataset because the subcategorized information of the brain physiome can be applied to variables in mathematical models [4].

Traditionally, researchers have evaluated drug toxicity using 2D *in vitro* and *in vivo* models [[5], [6], [7]]. 2D *in vitro* analysis enables high-throughput screening (HTS) of a few tens of thousands of candidates in a short time period, while *in vivo* models allow the analysis of drug absorption, distribution, metabolism, and excretion (ADME) [[8], [9], [10]]. However, a cell monolayer-based assay does not reflect the physiological microenvironment of the brain tissue. Furthermore, in *in vivo* analysis, the inherent genetic heterogeneity of animal models frequently limits the ability to predict human-specific adverse reactions to drugs. In addition, the 3R principles (replacement, reduction, and refinement) are gaining increasing recognition [11]. The 3R principles refer to (i) replacement of animal experiments with other approaches, (ii) reduction of the number of experimental animals, and (iii) alleviation of pain in experimental animals. Collectively, these principles suggest the need for an alternative to animal experimentation [12].

Of note, the evaluation and prediction of drug responses in brain tissue are more complicated than those in other organs. This is because brain tissue contains more cell types than other organs, and its structural complexity is greater than that of other tissues. Furthermore, the existence of the blood–brain barrier (BBB) limits the transport of small-molecule drugs and biopharmaceuticals to the brain, and hence, drug-associated adverse reactions in the brain tissue are difficult to analyze [13]. Indeed, no brain-targeting biopharmaceuticals have been approved to date, highlighting said difficulties [14].

Considering the above discussion, the emerging *in vitro* 3D platforms utilizing human cell sources are more attractive than their 2D *in vitro* and animal models because they can be used to monitor real-time drug effects and predict drug responses, with high similarity to those in humans, and are more reflective of human physiology than other models [15,16]. In general, the emerging *in vitro* 3D brain models are broadly classified into brain organoids and brain-on-a-chips, depending on whether the 3D cellular structure is formed with or without a scaffold. Each model type is associated with specific advantages in recapitulating the *in vivo* microenvironment [[17], [18], [19]]. On the one hand, brain organoids mimic the tight cell–cell interactions and region-specific physiology of the brain [[20], [21], [22]]. In contrast, brain-on-a-chip technology encapsulates various types of cells in a miniaturized 3D environment, and thus is able to recapitulate a physiological stimulus in the brain [[23], [24], [25], [26]]. In addition, these emerging *in vitro* 3D brain models can be used for research at various biological scales. In contrast, the findings of the existing 2D *in vitro* models cannot be readily translated to the organ level, and the those of the *in vivo* ones cannot be readily translated to the subcellular level [27,28].

In this review, we introduce a novel concept of the brain physiome, which can be used as a translational route to connect the emerging 3D *in vitro* brain models with *in silico* ones. For this purpose, we classified the components of the brain physiome into tissue- and cell-level components and summarized the approaches to study adverse drug reactions in the 3D *in vitro* brain models, with particular focus on the brain physiome. We also present the current limitations and future directions of the physiome concept and 3D *in vitro* brain models.

**Fig. 1:**
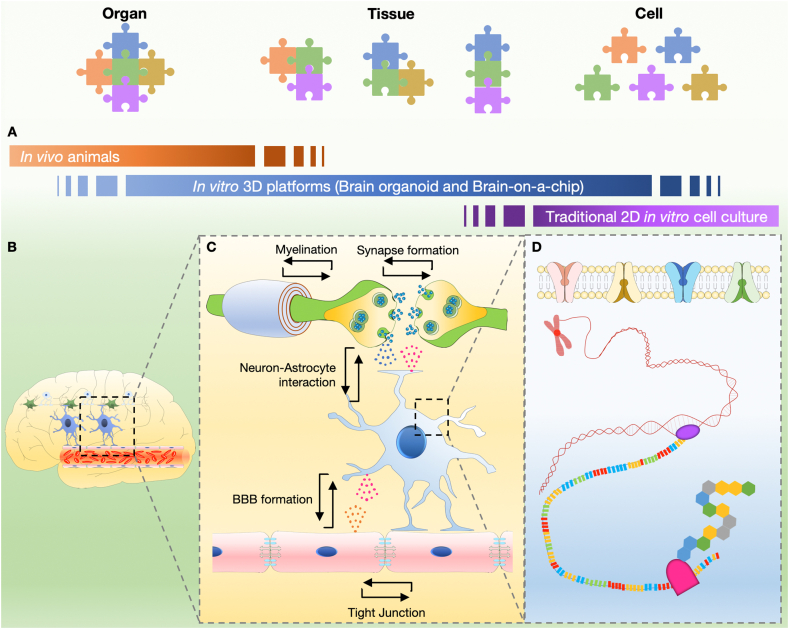
**Various levels of the brain as viewed through the physiome.** (A) The brain is an organ made up of tissues such as neural circuits, cerebrovascular cells, and supporting cells (e.g., astrocytes). (B) Each tissue consists of various cells with their characteristics, such as neurons, glial cells, and endothelial cells. Each type of cell interacts with either each other or between the same or different cells. (C) Each cell has a cell membrane, within which transcription and translation occur.

**Fig. 2:**
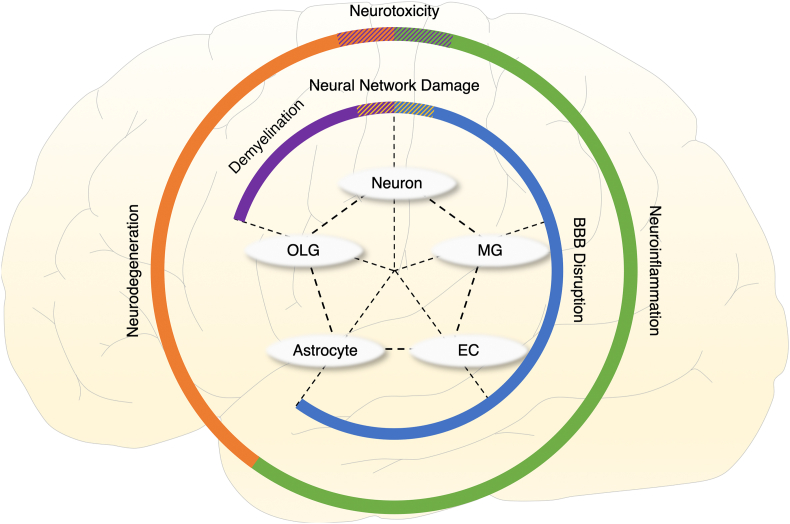
**Schematic of the correlation among various cells and tissue components in the brain physiome.** The neuron, the oligodendrocyte (OLG), the astrocyte, the endothelial cell (EC) and the microglia (MG) gather to form a neural circuit, myelination, the BBB and the immune system, leading to the prediction of neurotoxicity, neurodegeneration and neuroinflammation as drug side effects on the brain physiome.

Before explaining the brain physiome at the tissue and cell levels, we provide a schematic concept of *in vitro* 3D platforms that can cover the range of *in vivo* animal experiments and traditional 2D *in vitro* cell culture (Fig. 1A and Table 1, Table 2). The *in vitro* 3D platform can be used to study the interaction between various elements constituting the brain by decoupling them (Fig. 1B and C), and also to study the various types of cells constituting the brain, cultured in a 3D environment similar to the *in vivo* environment (Fig. 1D). To precisely predict the side effects of brain-targeting drugs, brain toxicity parameters should be classified into groups that can be explained by the brain physiome at the tissue and cell levels. At the tissue level, we address the interactions with neurons, brain endothelial cells, astrocytes, and oligodendrocytes, which construct brain tissue in the physiome (Fig. 2). At the cellular level, we focus on each cell's activity and metabolism, which emerges in the intracellular, cell membrane, and extracellular environments.

**Table 1:** Pathological brain toxicities on the tissue level and *in vitro* platforms for their analysis. Representative brain toxicity associated with the side effects of brain-targeted drugs is classified into neurotoxicity, neuroinflammation, and neurodegeneration. Using *in vitro* 3D brain platforms, such as brain organoid and brain-on-a-chip, the toxicity can be tested and the underlying mechanism of toxicity can be evaluated.

Toxicity type	Research focus	*In vitro* platform	Features of *in vitro* platform	Reference
Neurotoxicity	Neural network	Brain organoid	- Fused cerebral organoid with other brain region organoid - Vascularized brain organoid	[36] [39]
		Brain-on-a-chip	- Brain-on-a-chip consisting of endothelial cells, microglia, astrocytes, and neuroblastoma - Shows toxicity associated with organophosphate-based compounds	[58] [41]
Neuroinflammation	Blood–brain barrier	Brain organoid	- Brain spheroid consisting of astrocytes, pericytes, and brain endothelial cells - Receptor-mediated transcytosis simulated by angiopep-2 - Development of high-throughput permeability test platform	[115] [57]
		Brain-on-a-chip	- Consists of brain microvascular endothelial cells derived from iPSC - Shows high level of barrier function	[58]
Neurodegeneration	Myelination	Brain organoid	- Brain organoid consisting of neurons, astrocytes, and oligodendrocytes - Shows mature myelination - Demyelination occurs after lysolecithin treatment	[66] [32]
		Brain-on-a-chip	- CNS modeling (with oligodendrocytes) and PNS modeling (with Schwann cells) available - Promotes myelination by optogenetics - Development of high-throughput myelination platform	[77] [70] [73]

Abbreviations: CNS, central nervous system; iPSC, induced pluripotent stem cell; PNS, peripheral nervous system.

**Table 2:** Brain toxicity parameters on the cellular level, their features, and measurement methods.

Location	Cell type	Features	Measurement methods	Reference
Extracellular	Neuron	Neurotransmitter Concentration	- ELISA - Microelectrode biosensor	[92] [93]
		Dendritic spine density	- Imaging neurons expressing a fluorescent protein - Imaging immunostained neurons (neurolucida, F-actin)	[100]
		E/I balance	- RT-PCR or western blotting	[113]
Cell membrane	Endothelial cell	Permeability	- Use of fluorescent molecules with adjustable molecular weight - TEER - Imaging immunostained endothelial cells (ZO-1, occludin, claudin)	[116] [117]
	Neuron	Ion channel reactivity	- Imaging immunostained neurons (Nav 1.1, CACNA1B, KCNC2) - Electrophysiology - Gene expression heat map	[129]
		Receptor Overexpression or suppression	- Imaging immunostained neurons (GluA1, NMDAR1) - Electrophysiology	[126]
Intracellular	Glia	Glial cell reactivity	- Imaging immunostained glial cells (GFAP, CD68, Iba1, HLA-DR) - RT-PCR or western blotting	[134]
	ALL	Cell toxicity	- Live/dead assay - CCK assay - MTT assay	[135]

Abbreviations: CCK, cell-counting kit; E/I, excitation/inhibition; ELISA, enzyme-linked immunosorbent assay; MTT, 3-(4,5-dimethylthiazol-2-yl)-2,5-diphenyl-2H-tetrazolium bromide; RT-PCR, real-time polymerase chain reaction; TEER, transendothelial electrical resistance.

## 2 Tissue level

With considerable advances in 3D brain modeling platforms, the side effects and the therapeutic effects of drug candidates for neurodegenerative disorders can be predicted to accurately reflect the responses of human tissue. Below, we explain how *in vitro* 3D brain models can be used to test representative pathological side effects of drugs at the brain tissue level (i.e., at the level of neural network, BBB, and myelination). We also briefly mention drug testing at each level.

### 2.1 Neural networks

The brain is organized into multiple functionally heterogeneous regions, and it transmits and processes signals to/from each region to peripheral organs via interconnected neural networks. Adjacent neurons form synapses, with neurotransmitters released by presynaptic neurons and absorbed by postsynaptic ones. These signal transmission events control physiological and mechanical activities [29,30]. Therefore, protection of neural connectivity is crucial for correct signal transmission via neural networks and synapses, and for the maintenance of homeostasis in brain physiology. During chemotherapy, some chemotherapeutic agents exert adverse effects on the nervous system. Therefore, neurotoxicity is usually considered during the development of new drugs in *in vitro* and *in vivo* tests. Neurotoxicity is caused by exogenous molecules and endogenous metabolic products, such as nitric oxide (NO). These molecules eventually damage neurons and disrupt neural networks in the nervous system [31]. In this subsection, we focus on *in vitro* 3D neural network models that can be used for neurotoxicity testing.

**Fig. 3:**
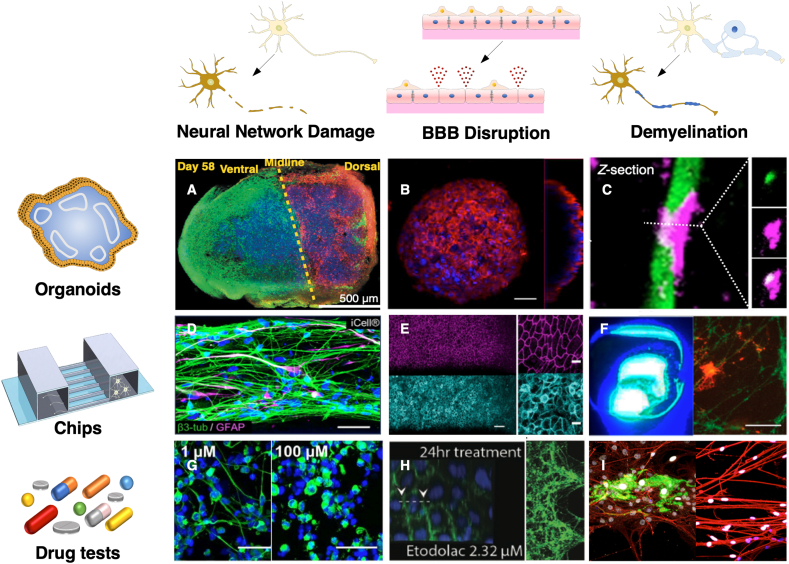
***In vitro* 3D brain models including brain organoids (upper lane, A-C) and brain-on-a-chips (middle lane, D-F).** The row shows the observable side effects in the *in vitro* brain system, whereas the column shows brain-mimetic platforms and the representative cases of drug testing. For example, the side effects of drugs can be estimated through the observation of neurotoxicity, neuroinflammation, and neurodegeneration (bottom row, G-I), and the *in vitro* neural networks used to treat neurotoxic molecules are shown in the left column (A, D and G); (A) Fused cerebral organoids (Reproduced from Bagley et al. with permission [36]. Copyright 2017, Springer Nature.), (D) Neuron and astrocyte networks in microfluidics (Reproduced from Wevers et al. under the terms of the CC BY license [42]. Copyright 2016, Springer Nature.), (G) Neurotoxin molecule (methylmercury) treatment (Reproduced from Wevers et al. under the terms of the CC BY license [42]. Copyright 2016, Springer Nature.). For assessing neuroinflammation (middle column, B, E, and H), an *in vitro* BBB was fabricated and used to treat anti-inflammatory drugs; (B) BBB organoids (Reproduced from Bergmann et al. with permission [57]. Copyright 2018, Springer Nature.), (E) BBB chips (Reproduced from Park et al. under the terms of the CC BY license [58]. Copyright 2019, Springer Nature.), (H) Anti-inflammatory drug (Etodolac) treatment. (Reproduced from Shin et al. under the terms of the CC BY license [60]. Copyright 2019, Wiley). To estimate neurodegeneration (right column, C, F, and I), *in vitro* myelination models are fabricated and used to treat neurotoxin drugs; (C) oligodendrocyte and neuron organoids (Reproduced from Marton et al. with permission [67]. Copyright 2019, Springer Nature.), (F) Myelination in microfluidics (Reproduced from Lee et al. with permission [69]. Copyright 2016, American Chemical Society), (I) Neurotoxin drug (tetrodotoxin) treatment (Reproduced from Hyung et al. with permission [70]. Copyright 2019, Wiley).

The integrity and structural complexity of brain organoids are higher than those of 2D brain cell monolayers [32]. Brain organoids can be classified into whole brain organoids and region-specific brain organoids [33,34]. Lancaster et al. generated whole cerebral organoids to closely implement endogenous development from human pluripotent stem cells (hPSCs) and developed cerebral cortex, ventral telencephalon, choroid plexus, and retinal identities within 1–2 months [35]. Such whole brain organoids are valuable in mimicking the *in vivo* physiology of the brain but have difficulty analyzing drug responses emerging between different regions precisely because of the heterogeneity in size and region-specificity or -coverage. However, the whole brain organoids are still useful in investigating the interactions between different brain regions. Alternatively, region-specific brain organoids, recently drawing more interest, possess great potential because they are relatively uniform in size and cellular composition. Single brain organoids do not fully reflect the regional brain identities because the anatomical structures of the native brain are more complicated than those obtained in *in vitro* brain models. Recently, fused cerebral organoids have been reported, beyond the construction of a single organoid, providing a highly advanced cerebral organoid model and representing the interaction between specific brain regions [36] (Fig. 3A). By fusing differentiated brain organoids, the authors generated a dorsal–ventral axis model exhibiting dorsal and ventral forebrain identities in each region.

Brain-on-a-chip technology has emerged as an alternative approach to brain organoids. Brain-on-a-chip has strong potential to recapitulate vascularized 3D tissue, which is essential for the prevention of necrosis in thick tissues and for providing physiological stimuli, such as appropriate fluidic conditions [37]. Furthermore, vascularization of the brain tissue is of primary importance because of the BBB structure, which prevents the entry of drugs into the brain [38]. From the cell–cell interaction perspective, the presence of vasculature-like structures results in enhanced functional maturation of organoids [39]. Therefore, a brain model with a functional BBB structure can closely predict the *in vivo* neurotoxicity of drugs. Integration of brain organoids and brain-on-a-chip has recently been attempted to better mimic the structural and functional characteristics of human brain tissue [40].

A few pioneering studies have utilized brain-on-a-chip to evaluate the neurotoxicity of various compounds. Organophosphate-based compounds (OPs), which are toxic substances found in pesticides, represent a major threat to civilian populations [41]. Koo et al. (2018) used a tetra-culture brain-on-a-chip model to show that OPs penetrate the BBB and rapidly inhibit acetylcholinesterase (AChE) activity. In addition, neurons and glia assessed neurotoxicity depending on methylmercury concentration [42] (Fig. 3D, G) and showed that the toxicity observed in the brain-on-a-chip model was correlated with the available *in vivo* data. This demonstrates that *in vitro* 3D brain models can potentially be used for neurotoxicity testing as an alternative to animal model-based experiments.

**Fig. 4:**
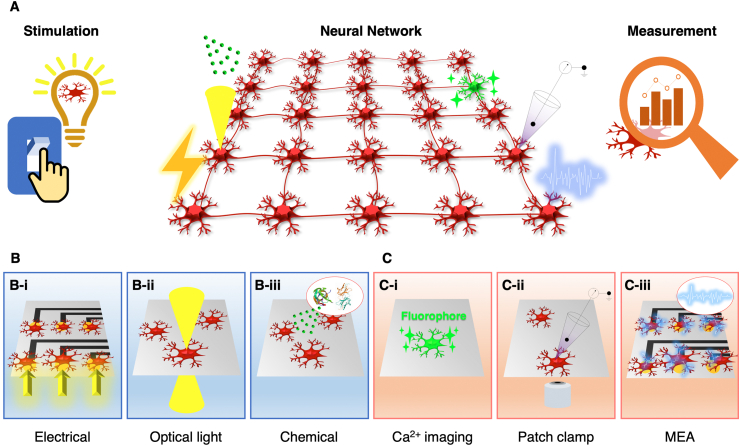
**Methods for stimulating the *in vitro* neural network and measuring their response.** (A) Schematic of the *in vitro* neural network and its utilization in the study by using stimulation and measurement methods. (B) Stimulation methods for *in vitro* neural network; electrical, optical light, and chemical stimulation (C) Measurement methods for neural activity; intracellular calcium ion signaling, patch-clamp, and microelectrode array (MEA).

Various techniques, such as electrical stimulation, optical light stimulation, and chemical stimulation, can stimulate neural networks locally to generate a transmitting action potential (Fig. 4A). The local stimulation technique can analyze signal transmission through neurons/synapses, which is the most critical characteristic of neural circuits. Furthermore, local stimulation can be used to study synaptic plasticity in neural circuits *in vitro*. Local stimulation in this context means that only a few neurons, or a few areas of neurons, are stimulated instead of the entire area in which neurons are being cultured. Electrical stimulation is achieved by applying an electric potential to the neurons in contact with the electrodes [43] (Fig. 4B–i). Optical light stimulation applies optogenetic techniques to control neurons by irradiating with optical light [44,45] (Fig. 4B–ii). Optogenetics is a technology that expresses photoreceptors on the cell membrane of neurons and can activate or inactivate neuronal activity using light of a specific wavelength [46]. Chemical stimulation involves the administration of a substance that depolarizes neurons. With the aid of microchannels, chemical stimulation can be performed only in specific zones of *in vitro* neural networks [47] (Fig. 4B–iii).

Long-range signal transmission can be observed and measured using various techniques (e.g., intracellular calcium ion live-imaging, patch-clamp, and multi-electrode array (MEA)) (Fig. 4A). It is essential to measure these signals to evaluate the functional connections of *in vitro* neural circuits. In a neuron, the intracellular concentration of calcium ions is 100 nM in the resting state, and the concentration of calcium ions increases to 1000 nM when the neurons are activated [48]. Given that the calcium ion concentration changes rapidly, real-time measurements are necessary. Intracellular calcium ion concentration indicators are tools for visualizing the frequency and amplitude of action potentials [49] (Fig. 4C–i). A patch-clamp is a tool developed to record single or multiple ion channel currents, and membrane potential, on the cell membrane. For a patch-clamp measurement, the micropipette containing the electrolyte solution was brought into contact with the cell membrane and a gigaseal was formed by applying negative pressure. Then the current flux through the membrane channels was recorded in the electrode [50]. The current measurement method in this state is called the whole-cell patch-clamp, and in this way, the neurons’ action potential can be measured [51] (Fig. 4C–ii). The MEA can identify the interconnections between different neurons in response to electrical stimulation. Using multiple electrodes and complementary metal-oxide-semiconductor (CMOS) technology, massive neural signals can be measured at a very high spatial and temporal resolution. This approach enabled analyzing the connectivity of neural circuits at the cellular level [52] (Fig. 4C–iii).

### 2.2 BBB

The human brain has a unique vascular structure called the BBB, which is composed of endothelial cells, pericytes surrounding the capillaries, and astrocytes interconnecting the neurons and the capillaries [53]. The BBB controls molecular transport between the blood and the central nervous system (CNS) and restricts access to potentially harmful solutes to the brain [54]. However, under pathological conditions, such as neuroinflammation, the BBB is disrupted, and various molecules penetrate the CNS. Neuroinflammation is caused by multiple factors, including injury, immune system imbalance, or infection. This pathological process includes cytokine release syndrome in humans. Therefore, incorporating a model of the human BBB in *in vitro* platforms has major consequences for evaluating whether a drug can reach the CNS under normal and pathological conditions.

A tri-co-culture system of brain endothelial cells, astrocytes, and pericytes was used to reproduce BBB properties and function in *in vitro* BBB models [55,56]. In these models, tight junctions are formed within the organoids with appropriate molecular transporters, and they can be used to model drug penetration across the BBB. Human BBB organoids can be used for rapid (within 1 day) screening of drug permeability across the human BBB by confocal fluorescence microscopy and mass spectrometry imaging [57] (Fig. 3B). In one study, a human BBB chip fabricated using a brain microvascular endothelium derived from human induced pluripotent stem cells (iPSCs) successfully recapitulated the barrier function, preventing antibody transport across the BBB [58] (Fig. 3E). Such BBB models may be used to validate the efficiency of drugs and therapeutic antibody delivery across the human BBB.

Drugs targeting neurons and microglia should be designed considering their interactions with the BBB [59]. Specifically, drug candidates targeting Alzheimer's disease (AD) and Parkinson's disease (PD) have to cross the BBB prior to exerting a therapeutic effect on degenerating neurons. To investigate the correlation between BBB dysfunction and AD, Shin et al. developed a human BBB chip composed of brain blood vessels and neurons. This model revealed increased BBB permeability, decreased expression of tight junctions, increased expression of matrix-metalloproteinase-2, increased reactive oxygen species levels, and deposition of β-amyloid (Aβ) peptides in the vascular endothelium in Alzheimer's disease (AD) [60] (Fig. 3H). Hence, this platform provides opportunities for screening new drugs that target AD and other neurodegenerative diseases.

Furthermore, another CNS barrier is the blood-cerebrospinal fluid (CSF)-barrier (B-CSF-B), which consists of the brain and epithelial barrier, and the choroid plexus (ChP). Similar to the BBB, B-CSF-B prevents free entry of toxic substances or drugs from the blood. While the BBB is formed by brain endothelial cells, astrocytes, and pericytes, B-CSF-B is established by choroid plexus epithelial cells. Pellegrini et al. showed that human ChP organoids with CSF production capability exhibited similar selectivity to small molecules observed in the ChP *in vivo*. Furthermore, the ChP organoids can predict the central nervous system (CNS) permeability of some small molecules such as bupropionyl, methotrexate, and vincristine [61].

### 2.3 Myelination

Adverse side effects of drugs can facilitate neurodegeneration, and demyelination is a well-known neurodegenerative pathological process [62,63]. The neural axon is covered with a myelin sheath, which insulates it and supports electrical conduction in neural circuits. Demyelination, a typical adverse drug side effect in neural tissues, refers to the peeling of the myelin sheath covering the axon. When the myelin sheath is damaged, the transmission of nerve signals is disrupted, which adversely affects body function [64].

Demyelination is a representative neurodegeneration-promoting process associated with the side effects of drugs. Consequently, *in vitro* myelination and demyelination models can be used to predict the side effects of drugs. Myelination and demyelination are observed in the CNS and the peripheral nervous system (PNS), depending on whether the myelin sheath is produced by oligodendrocytes or Schwann cells [65].

To date, only the CNS myelination model has been developed for organoids. Because of the lack of protocol optimization, the spontaneous emergence of oligodendrocytes is difficult to control in brain organoids generated using conventional methods. However, with the advent of new differentiation methods, organoids containing neurons, astrocytes, and oligodendrocytes have been developed [66]. Indeed, it has been confirmed that oligodendrocytes in organoids wrap around neuronal axons. In addition, myelin around the axon becomes more compact with culturing, indicating the maturity of myelination. After sufficient myelination in the organoid, demyelination can be simulated by treatment with lysolecithin, a substance known to induce demyelination [67] (Fig. 3C).

Both CNS and PNS models of myelination have been developed using brain-on-a-chip and nerve-on-a-chip respectively. In the CNS model using brain-on-a-chip, optogenetic stimulation and electrical stimulation promoted myelination of oligodendrocytes [68,69] (Fig. 3F). Similarly, in the PNS model using nerve-on-a-chip, optogenetic stimulation promoted myelination [70,71] (Fig. 3I). In addition, robust demyelination by drugs such as lysophosphatidylcholine (LPC), and remyelination by drugs such as benzatropine (Benz) or methylcobalamin (MeCbl), can be reproduced using nerve-on-a-chip [72]. Furthermore, platforms for high-throughput myelination experiments have been developed [73,74] and can be used to test the side effects of various drugs.

Finally, a unique platform has been developed that can simulate myelination and demyelination by oligodendrocytes alone, without neurons. The *in vivo* process of myelination does not require dynamic axon signaling. Instead, axon fiber's diameters play a pivotal role in regulating myelination *in vivo* [75,76]. The *in vivo* myelination occurs predominantly around axons with larger (e.g., >2 μm) diameter rather than small diameter. This characteristic was also reproduced with engineered microstructures. In oligodendrocyte culture using an electrospun polystyrene fiber platform, oligodendrocytes wrapped thick fibers (diameter: 2.0–4.0 μm) with a concentrical, multilayered sheath structure [77]; on the other hand, no wrapping occurred around thin fibers (diameter: 0.2–2.0 μm). This concentric wrapping morphology is characteristic of *in vivo* myelination. By applying the characteristics of oligodendrocytes, a conical micropillar array capable of HTS myelination was developed [78]. The conical micropillar had an upper diameter of 2 μm and a lower diameter of 50 μm. Subsequently, oligodendrocytes concentrically wrapped the micropillars and could be imaged readily on a microscope. Because the conical micropillar array can be mass-produced, HTS of myelination-related drugs is possible using this platform.

## 3 Cellular level

The safety evaluation parameters that are used for predicting the side effects of drugs at the cellular level are discussed below. We have classified them based on the region of interest, in which they can be analyzed, as extracellular, cell membrane, and intracellular regions.

### 3.1 Extracellular region

In the brain, the extracellular space is the space in the tissue outside of the cellular elements [79]. The extracellular space is filled with interstitial fluid containing various ions, proteins, and non-proteinaceous substances [80]. In this subsection, we focus on the synapse, the structure that plays a vital role in the brain's function. In the context of extracellular space, the synapse strictly refers to the synaptic cleft [81]; however, we have covered the entire synapse here. We also review neurotransmitters, which are substances that transmit signals in the synaptic cleft, the dendritic spines (the structures that make up the post-synapse), and the excitatory and inhibitory synapses.

The synapse is the structure that transmits signals between neurons. It consists of a presynaptic terminal, a postsynaptic one, and the synaptic cleft [82]. In general, the presynaptic terminal is located at the axon, the postsynaptic terminal is located at the dendritic spine, and the width of the synaptic cleft is 20 nm [83]. When an electrical signal arrives at the presynaptic terminal via the axon, a neurotransmitter is secreted from the presynaptic terminal to the synaptic cleft. When the neurotransmitter passes through the synaptic cleft and reaches the neurotransmitter receptor located at the postsynaptic terminal, it opens a channel in the cellular membrane of the postsynaptic neuron, thus generating an action potential [84].

Various types of neurotransmitters and chemicals transmit signals between neurons in the synaptic cleft. Glutamate is a representative, and abundant, excitatory neurotransmitter [85]. It is present in the extracellular space in the hippocampus at 15–20 μmol/L [86]. A change in the glutamate concentration upon drug administration is considered to be a side effect of the drug because it causes abnormal neuron function or even cell death [87]. For example, the reduction of glutamate concentration by anti-epileptic drugs results in slow information processing [88].

Typical methods for measuring neurotransmitter levels in the extracellular space include enzyme-linked immunosorbent assay (ELISA) and microelectrode biosensor measurements [89,90]. Microelectrode biosensors can be applied to living tissues and they allow real-time monitoring of neuron function [91]. In brain organoids, ELISA and microelectrode biosensors are both used to measure neurotransmitter concentrations [92,93]. In brain-on-a-chip, cells cultured inside the channel were first collected, and then ELISA was performed [58]. To measure neurotransmitter concentrations, the sensing tip of a conventional microelectrode biosensor is generally inserted into the tissue or culture medium. The electrodes can be integrated within the brain-on-a-chip platform, which enables real-time monitoring of cellular growth and death during the entire culturing period [94]. Furthermore, a brain-on-a-chip designed to expose a hydrogel containing 3D-cultured cells to the external environment has been recently developed [95]. Using this approach, a microelectrode biosensor in the form of a sensing tip can be used, even in a brain-on-a-chip.

Neurotransmitter receptors are located at the postsynaptic terminals. Most postsynaptic terminals are located in the dendrites and form unique dendritic spine structures [96]. Dendritic spine density falls within a specific range for a healthy neuron, and aberrant dendritic spine density induces brain dysfunction [97]. For example, dendritic spine density is increased in autism spectrum disorders (ASD), and reduced in schizophrenia and AD [98]. This is critical, as dendritic spine density can change in response to the administered drug [99]. Dendritic spine density is evaluated by imaging fluorescent protein expressing neurons, or immunostained ones [100,101]. Electron microscopy can also be used to measure the dendritic spine density in brain organoids, although data evaluation is challenging because of the narrow field of view afforded by this technique [102]. Distinguishing dendritic spines of individual neurons using conventional methods that rely on the expression of fluorescent proteins or immunostaining is also challenging because of the high neuron density in brain organoids. Therefore, identifying changes in dendritic spine density in response to drugs in brain organoids is currently difficult. In contrast, in the brain-on-a-chip, neurons are relatively sparse, and dendritic spine density can be measured by fluorescence imaging [103].

Furthermore, synapses are classified as excitatory or inhibitory synapses. A signal crossing the excitatory synapse increases the activity of the receiving neuron; conversely, a signal crossing the inhibitory synapse decreases the activity of the receiving neuron [104]. The balance between these two types of synapses (excitation/inhibition (E/I) balance) is essential for determining the activity of a neural circuit. When this balance is disrupted, brain activity increases or decreases accordingly [105,106]. For example, one of the pathologies of ASD is associated with increased E/I balance [107]. The E/I balance can either be analyzed on a single neuron or a neural network scale [108]. At the single neuron level, excitatory and inhibitory synapses can be analyzed in various ways. The type of synapse can be identified using the morphology of individual synapses [109]. Electrophysiological analysis can also compare excitatory postsynaptic currents (EPSCs) and inhibitory postsynaptic currents (IPSCs) [110,111]. In addition to measuring the E/I balance at the single neuron level, which was recently developed, there is a method to count the number of synapses by showing different fluorescence of inhibitory synapses and total synapses through transfection [112]. However, it is difficult to study the E/I balance on a large scale using the methods described above, such as the neural network,. The E/I balance analysis of neural networks can be performed using real-time polymerase chain reaction (RT-PCR) and western blotting, using a marker suitable for each synapse type [113]. E/I balance analysis methods for these neural networks facilitate the analysis of the E/I balance before and after drug injection in both brain organoids and brain-on-a-chip.

### 3.2 Cell membrane

The cell membrane contains various ion channels, receptors, and transmembrane proteins that exchange ions and other molecules between cell compartments [114]. Furthermore, it contains several junctions, such as tight junctions and gap junctions, which help in integrating adherent cells, and in constructing molecular transport barriers.

As mentioned before, BBB permeability is a critical parameter for evaluating BBB toxicity in *in vitro* studies. The permeability can be evaluated by measuring endothelial cell function and marker expression. To measure endothelial cell function, some model molecules (i.e., albumin and dextran), or drugs are introduced into an engineered blood vessel, and their leakage or transport is monitored [115]. In addition, transendothelial electrical resistance (TEER) is used to measure the tightness of a cellular barrier in *in vitro* systems [116]. If the endothelial cells are tightly connected, the TEER is low. Tight junction proteins, such as zonula occludens-1 (ZO-1), occludin, and claudin, expressed by endothelial cells, are standard markers of endothelial cell integrity. They are also used as markers of BBB toxicity [117]. The expression of tight junction proteins can be downregulated depending on the concentration of the administered drug. Recently, BBB organoids that reproduce the expression of transporters and drug efflux pumps *in vivo* have been used to predict BBB function in response to drug concentration [57]. Similar to BBB permeability, B-CSF-B permeability, a parameter of CNS permeability, was analyzed by observing the fluorescence intensity of ZO-1 and the transport of fluorophore-conjugated dextran [61].

Furthermore, in some cases, pathological stimulation and drug treatment activate (or inactivate) ion channels and receptors [118,119]. For example, ion channels are activated in individuals with Dravet syndrome, also known as severe myoclonic epilepsy in infancy (SMEI), which is an autosomal dominant genetic disorder. Mutations in the *SCN1A* gene induce a decrease in sodium currents and impair the excitability of GABAergic interneurons that transmit γ-aminobutyric acid (GABA) in the hippocampus, resulting in epilepsy [120]. Most anti-epilepsy drugs used to treat Dravet syndrome inactivate sodium ion channels and limit rapid excitation in the brain [121]. ASD is a neurodevelopmental disorder causing difficulties with social communication and interaction, and repetitive behavior in early childhood [122]. Although ASD has heterogeneous pathogenesis, various ASD models with *shank* gene mutation show NMDAR dysfunction [123,124]. Therefore, some medications for treating autism should target this receptor [125]. In the clinic, individuals harboring genetic variants of NMDAR display impaired social interactions and repetitive behaviors. Because no currently used medication can successfully treat ASD, mechanisms of ASD should be thoroughly analyzed, and candidate drugs that modulate NMDAR function should be identified.

Electrophysiology is a useful tool for monitoring changes in the current in membrane channels and receptors after drug treatment. The expression of ion channels and receptors can be determined by immunofluorescence staining of proteins, such as Nav 1.1 (Na^+^ channel), CACNA1B (Ca^2+^ channel), KCNC2 (K^+^ channel), AMPA receptor (GluA1), and NMDAR1 [126]. In the case of calcium ions, fluctuations in calcium concentrations can be observed under a microscope [127].

### 3.3 Intracellular region

The central dogma states that within a biological system, DNA is a template for RNA synthesis (for transcription), which in turn acts as a template for protein synthesis (for translation) [128]. Thus, it explains how genetic information is transferred and then expressed as a phenotype. Therefore, abnormalities in gene and protein expression can be determined based on the genetic information.

Advances in molecular technologies have enabled the analysis of the full spectrum of genome sequences in humans, including healthy individuals and patients. Knowledge of patient genomic information is beneficial for understanding disease mechanisms and predicting the therapeutic effects of drugs in idiopathic diseases. For instance, although many genetic background factors cause PD, the LRRK2-G2019S mutation has received much attention. It induces neuronal abnormalities, even in patients with mutated LRRK2 who display a mild phenotype [17]. The progression of this genetic mutation-dependent disease highlights the need for precision medicine.

At the genomic and transcriptimnic landscape, whole-genome sequencing can be used to determine the contribution of the genetic background to neuronal abnormalities [129]. RNA-seq is a commonly used approach to bulk and single-cell transcriptome profiling that reveal the high-throughput and systemic mapping of gene expression in brain tissues or individual cell. [130,131].Recent single-cell messenger RNA sequencing (scRNA-seq) initiated a new era of molecular studies that uncover the diversity and cellular identity of brain cell types. By combining many computational techniques used for electrophysiology, these transcriptomic data thus provide a better understanding of functionally distinct brain cell types. Furthermore, single-cell multi-omics, including epigenomics, trancriptomics and proteomics, can also provides informative datasets for computational analysis. [132,133].

At the cytokine or growth factor levels, they are synthesized intracellularly and then secreted. Hence, neuroinflammation can be evaluated based on the secretion of proinflammatory cytokines by cells, as measured by ELISA or cytokine arrays [134]. Furthermore, cytotoxicity of drug treatment can be predicted by using a live/dead assay, a cell counting kit (CCK) assay, or a 3-(4,5-dimethylthiazol-2-yl)-2,5-diphenyl-2H-tetrazolium bromide (MTT) assay [135].

## 4 Preclinical applications combined with imaging technologies and *in silico* modeling

### 4.1 Current limitations for collecting *in vitro* database

**Fig. 5:**
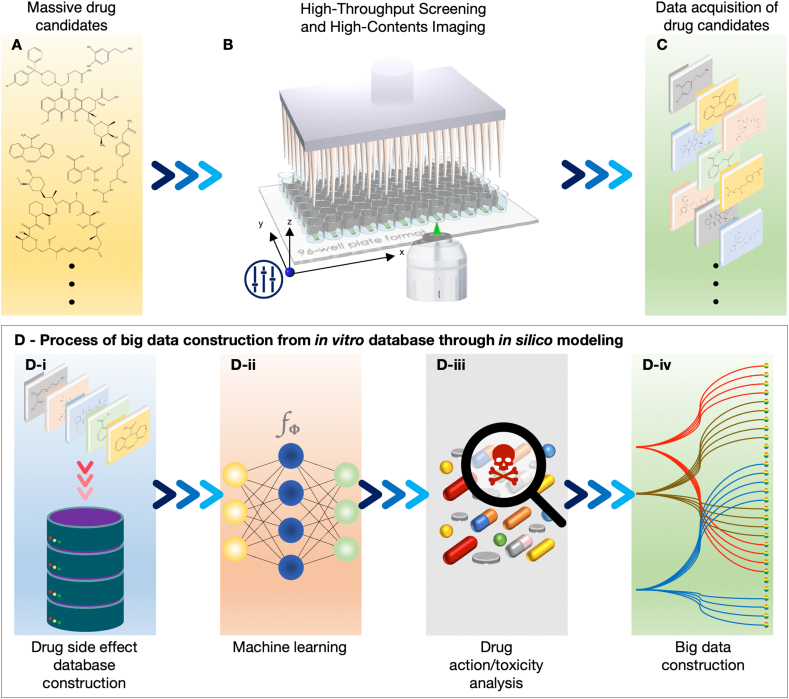
**A process for collecting results regarding *in vitro* drug side effects and providing information about drug action and toxicity, to simulate computational modeling combined with imaging technologies**. (A–C) Firstly, the massive drug candidates and side effects of the drugs need to be collected for constructing a drug side effect database using high-throughput and high-contents imaging. (D-i and D-ii) Then, the database is classified and reproduced to match the drug action/toxicity analysis through machine learning applications. (D-iii and D-iv) Finally, the computational modeling and simulation build up the big data predicting the side effects of target drug candidates.

For preclinical approaches of *in vitro* 3D brain models, we need to handle massive amounts of experimental data from thousands of drug candidates (Fig. 5A and C). Because a large number of images are required to produce data, the use of advanced imaging technologies, known as high-content screening (HCS) and HTS (Fig. 5B), is required. HCS is a method for analyzing the adverse reactions of various drugs in living cells at high temporal and spatial resolutions [136]. For HCS, experimental tools and techniques have been utilized to extract large amounts of complex information from tissue-mimetic platforms [137]. To date, organoids and organs-on-a-chip that recreate *in vivo*-like microphysiology are potential candidates for HCS.

In contrast, HTS is a fast and economical method of identifying adverse reactions of drugs using laboratory automation [138]. The goal of HTS is to minimize the time and the expenses of screening large numbers of drug libraries [139]. Therefore, to screen the adverse effects of numerous drug libraries in a time-efficient and reliable manner, it is necessary to develop organoids and organs-on-a-chip applicable to HCS and HTS. This development should proceed in two directions: i) development of a system for mass production with low variability and ii) development of a system capable of analyzing numerous inputs in parallel.

### 4.2 Mass-production and advanced analysis technologies for collecting *in vitro* database

Mass-production of brain organoids should be focused on reducing sample-to-sample variability. Organoids fabricated using low-attachment plates exhibit uncontrollable size and floating morphology and are unsuitable for reliable drug screening [140]. However, as the method to fabricate a microcavity array in a well-plate was developed, it became possible to manufacture organoids of uniform size, exhibited increased growth speed, reduced time to maturity, and had a fixed location [141,142]. Another advantage of microcavity arrays is that automated systems can replace traditional labor-intensive manual experiments such as cell seeding, medium exchange, drug exposure, and imaging [141,143]. This mass-production system is also being introduced in the field of brain organoids [[142], [143], [144]]. Mass-produced BBB organoids maintain the property of showing low permeability to macromolecules [144]. The mass-produced midbrain organoids maintain synchronized neural activity [143]. Mass-produced retinal organoids maintain the expression of photoreceptors [142]. The strategies for mass-production of brain-on-a-chip focus on a simple microfluidic device design combined with traditional labware [73]. This is because conventional labware, such as 96-well plates, is already available for HTS [145]. To this end, it is necessary to change the material used for the production of brain-on-a-chip, from polydimethylsiloxane (PDMS) to polystyrene (PS), and manufacture it by injection-molding fabrication. PS is more suitable for mass production and long-term storage than PDMS in terms of material properties [146].

High-resolution imaging and electrophysiology must be available for the analysis of brain organoids and brain-on-a-chip to allow for the evaluation of drug responses. The limit of the visual depth of conventional confocal microscopy is approximately 1 mm due to light scattering, which is relatively small compared to the diameter of the brain organoid, which is a few millimeters in diameter [147]. Therefore, the internal structure of the brain organoid cannot be identified using conventional imaging techniques. However, a tissue-clearing technique was recently introduced and opened a way to overcome the limitations of deep-tissue imaging [148], which allowed for observing the internal structure of the brain organoid with confocal and two-photon microscopy. Furthermore, light-sheet microscopy capable of high-throughput imaging of transparent brain organoids has been developed. Conventional confocal microscopy is not suitable for high-throughput imaging because it has limitations in high-speed imaging due to its point-based illumination and scanning approach. In contrast, light-sheet microscopy illuminates and scans the organoids or 3D tissue construct in a plane-by-plane manner so that the imaging speed dramatically increases, making it suitable for high-throughput imaging [149]. By applying tissue-clearing technology and light-sheet microscopy simultaneously, it is possible to image a whole-brain organoid with single-cell resolution in approximately 15 min [150]. In the case of brain-on-a-chip, fusing this approach with the use of traditional labware is helpful for high-throughput imaging. Incorporation of conventional labware, such as well plates in conjunction with automated microscope systems, in experimental design can accelerate image-based analysis [73].

In electrophysiology, high-throughput analysis can be achieved by transferring the brain organoid onto a commercially available MEA and culturing further [151]. In the case of brain-on-a-chip, commercially available MEAs can be combined with a microfluidic device, enabling high-throughput electrophysiology analysis [152].

### 4.3 Processing collected big data from *in vitro* database through *in silico* modeling

The number of studies based on the brain physiome for *in silico* modeling is limited, and the standards for data collection based on the brain physiome have not yet been established. The lack of data for the brain physiome hinders the development of effective *in silico* prediction models. Nonetheless, BBB permeability is the most well-collected database for the brain physiome. BBB permeability databases have been reported in several studies [153,154]. For *in silico* models predicting BBB permeability, numeric values, such as logBB or logPS, were used to describe BBB permeability. The prediction models were divided into two categories: classification and regression models. Classification models are binary classifiers for BBB permeability, and regression models are used to predict a numeric value representing BBB permeability, such as the logBB value or logPS value. With the advances in machine learning techniques, prediction tools have used different machine learning methods, such as support vector machine (SVM) and random forest [[155], [156], [157]]. Recently, combined machine learning models and consensus models have also been developed to improve prediction performance [[158], [159], [160]].

Most *in silico* brain physiome models suffer from a lack of data and imbalanced data problems. Even for the BBB permeability models, regression models show limited performance owing to the relatively small datasets, and quantitative models have the problem of imbalanced data [158]. For this reason, most brain physiome studies have focused on the construction of proper datasets. Jiang et al. collected a large number of mouse toxicity datasets to build a chemical neurotoxicity prediction model [161]. To predict *in vitro* neurotoxicity induced by nanoparticles, Furxhi and Murphy created a dataset containing nanoparticle physicochemical properties, exposure conditions, and *in vitro* characteristics from experimental neurotoxicity data from multiple studies [162]. Jamal et al. proposed constructing a neurological adverse drug reaction prediction model to arrange the side effects of drugs using biological data from DrugBank, chemical data from PubChem, and phenotypic data from the SIDER database [163]. Oversampling methods such as the synthetic minority oversampling technique (SMOTE) have been used in several studies to handle imbalanced data, which show improvement in model performance [158,159,162,163].

In contrast, a cardiac physiome database is available for predicting the cardiac toxicity of drugs and has already been constructed using *in silico* tools [4,164,165]. Hence, below, we explain the concept of the brain physiome based on the already established concept of the cardiac physiome, with the aim of extending the hierarchical elements of the brain physiome, to set up the standards for *in silico* modeling.

Computational modeling and simulation can be used to predict various biological phenomena, such as cardiac electrophysiology, based on experimental data [166,167]. For instance, a comprehensive *in vitro* proarrhythmia assay (CiPA) represents the relationship between the outcomes of *in vitro* analysis of drug effects (input data), and those from *in vivo* studies (output data) [168]. In this manner, assessing drug effects on multiple ion currents involves monitoring the effects on human cardiac myocytes *in vitro* and human electrocardiogram *in vivo*, in phase I clinical trials. Similar to cardiac modeling, the effects of brain-targeting drugs may be predicted using the brain physiome by simulated *in silico* brain modeling. First, drug candidates are selected and their side effects, such as brain toxicity, are examined using *in vitro* 3D brain models (Fig. 5 D-i). Then, an *in vitro* database is constructed to classify the data according to any tendencies, resulting in the construction of big data (Fig. 5D–ii). Next, the selected drug candidates are used as an input, and the predicted drug action and toxicity are used as an output in a drug side-effect database using machine learning (Fig. 5D–iii and 5D-iv).

## 5 Summary and outlook

**Fig. 6:**
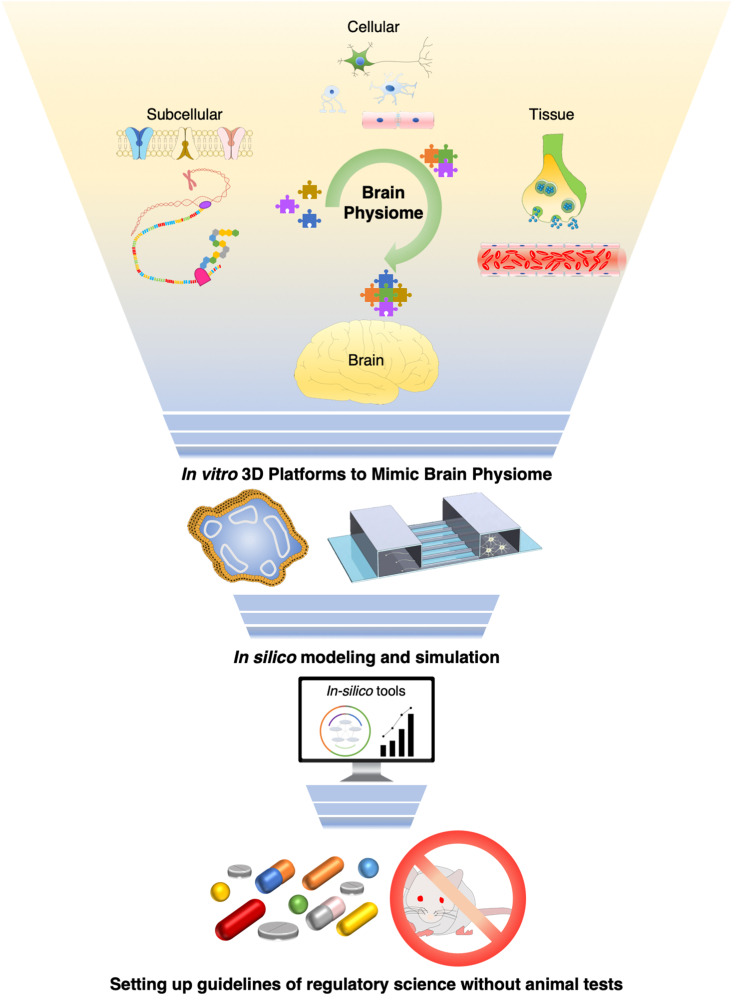
**The brain can be explained from various perspectives using the physiome.** These brain features viewed from various perspectives can be reproduced on high-technology *in vitro* platforms, brain organoids, or brain-on-a-chip. Furthermore, by providing data about various drug side effects, obtained from high-technology *in vitro* platforms, to an *in silico* tool, it will be possible to conduct high-throughput discovery of drug side effects and reduce laboratory animals' sacrifice.

Although newly developed biopharmaceuticals are effective against severe disorders, the analysis of their safety is challenging. Analysing adverse reactions to drugs targeting the brain is even more challenging because of the structural complexity of the brain and the inability to monitor deep tissue. To understand the complex characteristics of the brain, it is necessary to introduce the concept of the physiome. Considering the genetic heterogeneity of the physiome of the animal brain and that of the human brain, an *in vitro* brain model based on human cells that can recapitulate the brain physiome is required. Brain organoids and brain-on-a-chips are suitable solutions for these unmet needs in the field. Furthermore, to predict and minimize adverse reactions to novel drugs, it is necessary to introduce *in silico* tools that rely on computational resources to analyze the beneficial or adverse effects of chemicals or drugs [169,170]. To establish reliable *in silico* models, it is necessary to bridge the data obtained from *in vitro* brain models, such as brain organoids and brain-on-a-chips, with an *in silico* database. The standardization of input and output is also important for the evaluation and prediction of the side effects of newly developed drugs in a systemic manner. Once that is established, we anticipate that the number of *in vivo* experiments needed to predict the safety of biopharmaceuticals will be minimized (Fig. 6).

## Author contributions

S. Bang, Y. Seo, N. Choi, and H. N. Kim conceived the entire review and wrote the manuscript. Y. Seo and S. Bang surveyed all the literature and categorized reports into the hierarchical levels of the brain physiome. J.-H. Eom, H. N. Kim, P. Kim, and Y. Jeong conceptualized the scenario of the brain physiome-based prediction and evaluation of drug toxicity using *in silico* and *in vitro* models, respectively. J. Son and D. Kim reviewed reports related explicitly to *in silico* models and wrote the manuscript. All the authors reviewed the manuscript, provided constructive corrections and feedback toward strengthening the rationales of this review, and approved the final version of the manuscript.

## Declaration of competing interest

The authors declare that they have no known competing financial interests or personal relationships that could have appeared to influence the work reported in this paper.
